# Struma ovarii: role of imaging?

**DOI:** 10.1007/s13244-013-0303-3

**Published:** 2013-12-20

**Authors:** Martine I. Dujardin, Priti Sekhri, Lindsay W. Turnbull

**Affiliations:** 1 Centre for MR Investigations, University of Hull in association with Hull York Medical School, Anlaby Road, Hull, HU3 2JZ East Yorkshire UK; 2 Hull and East Yorkshire Hospitals NHS Trust, Histopathology Department, Hull Royal Infirmary, Anlaby Road, Hull, HU3 2JZ UK; 3 Centre for MR Investigations, Hull Royal Infirmary, Anlaby Road, Hull, HU3 2JZ UK

**Keywords:** Struma-ovarian tumour, Imaging, MRI, Cystic

## Abstract

As clinical features in struma ovarii patients in the absence of thyrotoxicosis are generally non-specific and resemble ovarian malignancy, preoperative radiological diagnosis becomes all the more relevant in order to avoid ovarian cancer type surgery (including bilateral salpingo-oophorectomy, hysterectomy, omentectomy and occasionally appendectomy) for this usually benign and rare ovarian mass. As struma ovarii is an uncommon entity, it is all the more important to perform state-of-the-art magnetic resonance (MR) imaging, including high-resolution imaging and diffusion-weighted imaging. The goal of this review paper is to give an update of the key findings of both benign and malignant struma ovarii and to present an unusual case of a purely cystic ovarian struma.

*Key Points*

• *Clinical features in struma ovarii are generally non*-*specific and resemble ovarian malignancy*.

• *Pre*-*operative radiological diagnosis is important to avoid ovarian cancer type surgery* (*bilateral salpingo*-*oophorectomy*, *hysterectomy*, *omentectomy and occasionally appendectomy*).

• *State*-*of*-*the*-*art MR imaging might help to characterise this unusual ovarian mass*.

• *Struma ovarii can occasionally present as a purely cystic lesion*.

• *However, the role of imaging to identify struma ovarii preoperatively remains limited*.

## Introduction

Struma ovarii is a very rare and usually benign ovarian tumour that was first described in 1889 by Boettlin [1], who observed the presence of thyroid follicular tissue in ovaries. It accounts for 0.3–1 % of all ovarian tumours [2] and for 3 % of all mature teratomas [3]. Struma ovarii is the most common form of monodermal teratoma and is characterised by the presence of macroscopically and histologically detectable thyroid tissue containing variable-sized follicles with colloid material [2]. Thyroid tissue is observed not uncommonly in 5–15 % of all dermoid tumours, but in order to qualify as a struma ovarii, the proportion of thyroid tissue present must comprise more than 50 % of the overall tissue [4].

Struma ovarii presents as a multi-cystic mass with a peak incidence in the 5th decade of life and peak age at presentation of 50 years [5]. When uncomplicated, the clinical manifestations of struma ovarii provide only limited information and often overlap with those of other diseases. As most patients are asymptomatic or present without specific features, diagnosis is often delayed until the development of symptoms related to ovarian torsion, hyperthyroidism or ascites. A study performed by Yoo et al. in 2008 [6] states that the most common initial symptoms at presentation are abdominal pain and distension or a palpable mass. Vaginal bleeding has also been described. Cases presenting with facial flushing and palpitations are rare [3]. Such clinical signs of thyrotoxicosis are reported in only about 5 % of struma ovarii patients [7, 8].

Meigs’ syndrome is characterised by the presence of ascites and pleural effusion in association with a benign ovarian tumour, typically an ovarian fibroma. Pseudo Meigs’ syndrome, on the other hand, is defined by the presence of ascites and a pleural effusion in association with either a mature teratoma, leiomyoma, cystadenoma or an ovarian malignancy [9]. In both syndromes, the ascites and pleural effusion resolve after the ovarian lesion is excised. Pseudo Meigs’ syndrome is occasionally encountered in benign struma ovarii [10, 11]. According to several authors [12–15], a variable occurrence of ascites in patients with struma ovarii, ranging from 17 % to 33.3 %, has been observed and it is thought that struma ovarii should be included in the differential diagnosis of a pelvic mass that presents with ascites, hydrothorax and elevated CA125 tumour marker [16].

However, CA125, the widely accepted tumour marker of ovarian carcinoma, is of little clinical value in struma ovarii patients as it can be elevated in both benign and malignant subtypes and because it is not consistently elevated even in malignant cases [6]. It has been postulated that an increased level of CA125 in struma patients is not a direct consequence of the presence of a tumour as such, but rather a secondary effect due to the presence of ascites [17].

## Imaging findings in struma ovarii

**Fig. 1:**
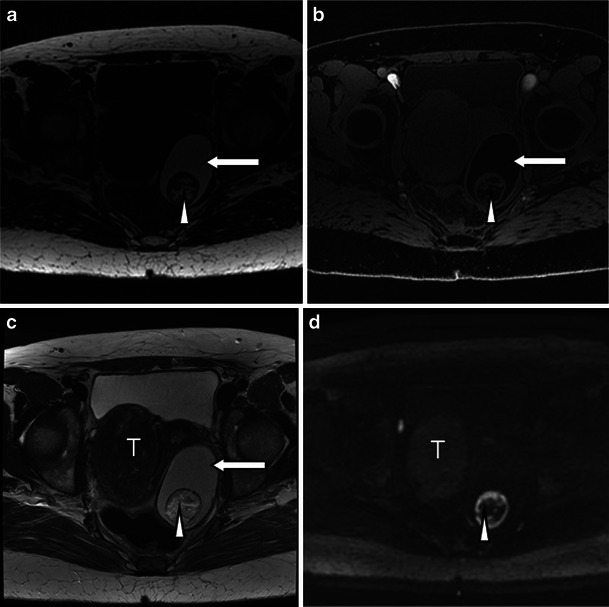
Three-tesla MR images of a mature teratoma or dermoid cyst arising from the left ovary in a 53-year-old patient. The fatty component in the lesion is hyperintense on the 3D-LAVA fat-only T1-weighted image (**a**) and hypointense on the water-only T1-weighted image (**b**) (*arrows*). **c** T2-weighted high-resolution image shows the fatty component within the lesion to be hyperintense (*arrow*). The nidus itself contains a fatty component that is also hyperintense on 3D-LAVA fat-only T1-weighted image (**a**) and hypointense on the water-only T1-weighted image (**b**) (*arrowheads*). **d** Diffusion-weighted image using a *b* value of 1,200 s/mm^2^ shows a high signal intensity rim representing restricted diffusion from sebaceous material surrounding the nidus and punctuate areas of restricted diffusion scattered throughout the nidus (*arrowhead*). The pedunculated uterine fibroid (*T*) at the level of the right lateral aspect of the uterus presents with typical hypointensity on T2 (**c**) without associated restricted diffusion (**d**)

Struma ovarii is a rare type of mature teratoma, but its imaging features are rather distinct. Whilst immature teratomas are known to be predominantly solid with small foci of fat, mature cystic teratomas or dermoid cysts are known to be predominantly cystic, present as a fat-containing mass and are often associated with calcifications as well as an enhancing nodule-forming soft-tissue component [3]. Diagnosis of uncomplicated teratomas using computed tomography (CT) and magnetic resonance (MR) imaging is fairly straightforward because both techniques are highly sensitive in the detection of fat within the tumour, presenting as negative attenuation on CT [7]. On MRI, three methods have been used to distinguish the fatty contents of the mature cystic teratoma from those of endometriomas or other haemorrhagic cysts. First, chemical shift artefact in the frequency encoding direction can be used to detect fat and to distinguish fat from haemorrhage [18]. Second, gradient-echo imaging, using an echo time in which fat and water are in opposed phases, can demonstrate fat–water interfaces and mixtures of fat and water [19]. Third, sequences with frequency-selective fat saturation will suppress the high signal of the intralesional fat in teratomas and help distinguish them from haemorrhagic lesions [3]. MR imaging with this latter technique allows accurate differentiation between teratomas and haemorrhagic cysts and is preferable to the other techniques [3]. The key features of a mature teratoma using water-only and fat-only T1 technique and diffusion-weighted imaging are demonstrated in Fig. 1. Of note is the restricted diffusion present in the sebaceous material within the cyst (Fig. 1).

Unlike the most common types of teratoma, namely mature and immature subtypes, struma ovarii does not demonstrate lipid material on either CT or MRI [3]. When struma ovarii is accompanied by foci of fatty tissue, they can be considered as areas of dermoid cyst associated with struma ovarii [20]. In these circumstances, the presence of dermoid cyst can be a strong clue to suspect struma ovarii [21].

### Scintigraphy

**Fig. 2:**
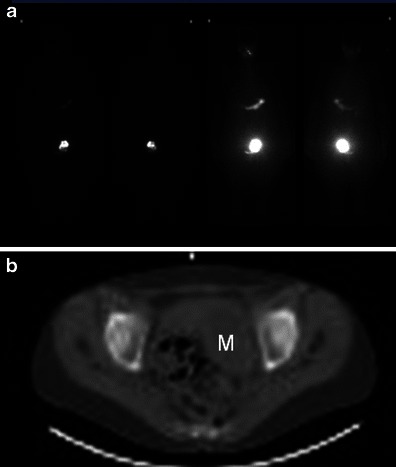
**a** Whole-body I-123 imaging shows significantly increased I-123 uptake within the left hemi-pelvis in a 61-year-old woman with a history of thyrotoxicosis treated with radioiodine treatment twice over the last 30 years, who ultimately had a thyroidectomy which showed Graves disease but who remained thyrotoxic post surgery. **b** The area of increased uptake within the left hemi pelvis corresponds on the low-dose CT to an 8-cm left-sided pelvic mass (*M*) in keeping with a confirmed struma ovarii

Scintigraphy performed with either iodine-123 or iodine-131 [22] is useful for diagnosing a hyperfunctioning struma ovarii on the basis of higher uptake of the radionuclide by the ovarian mass compared with the thyroid gland (Fig. 2).

### Ultrasound

**Fig. 3:**
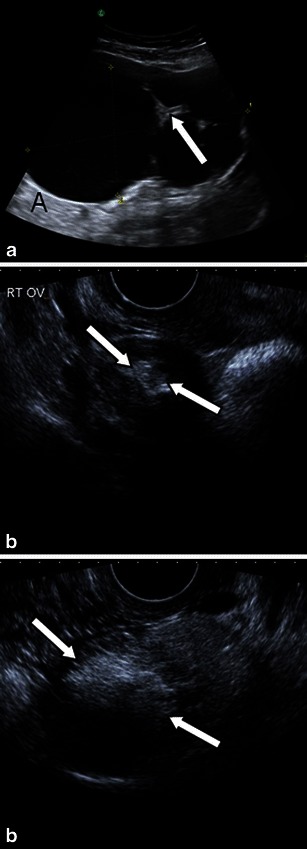
Difference in ultrasound features between struma ovarii and ovarian dermoid cyst. **a** Predominantly cystic struma ovarii typically mimicking an epithelial ovarian tumour (either cystadenoma or borderline ovarian tumour) in the right ovary of a 51-year-old patient presenting with non-specific abdominal cramps. Some thicker septations are seen (*arrow*) between otherwise cystic areas presenting with anechoic fluid and posterior acoustic enhancement (*A*).*Right* 1.5-cm (**b**) and *left* (**c**) 5.1-cm dermoid cyst in a 24-year-old woman. Both lesions on ultrasound have the typical pattern of high attenuation within the cyst (*arrows*) representing its fatty component

Whilst a mature teratoma or dermoid cyst presents on ultrasound as a cystic lesion with a densely echogenic tubercle (termed a Rokitansky nodule or nidus), which projects into the cystic lumen [3], as well as associated sound attenuation due to sebaceous material and hair within the cyst cavity or recognisable fluid–fluid levels from the sebum floating above aqueous material [3, 23], the ultrasound features of a struma ovarii are quite indistinct (Fig. 3). As struma ovarii presents with a variety of non-specific appearances and usually manifests as a multilocular cystic ovarian mass with solid components of various amounts, the ultrasound typically demonstrates these non-specific heterogeneous solid cystic features [3].

### CT

**Fig. 4:**
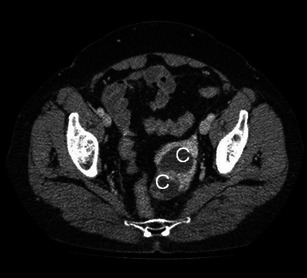
Venous phase enhanced CT image in a 69-year-old woman shows a well-delineated lesion within the left hemi-pelvis. The peripheral solid component of the lesion shows marked contrast uptake while two well-delineated cystic areas of low signal intensity (*C*) are seen centrally within the cyst. At histopathology this proved to be a struma ovarii with a papillary microcarcinoma component

Although findings are generally non-specific, CT demonstrates the complex appearance with multiple cystic and solid areas, reflecting the gross pathological appearance of the struma. Shen et al. [24] found high-density cysts with CT values ranging from 58 to 98 Hounsfield units (HU) in 68 % of their cohort of 12 cases. They hypothesised that the appearance of such high-density cysts in a pelvic mass, especially when greater than 90 HU [24], are caused by both thyroglobulin and thyroid hormones in the ovarian follicular thyroid tissue attenuating the X-ray beam. Shen et al. [24] proposed that these high-density cysts might be a characteristic feature of struma ovarii on CT (Fig. 4). Ikeuchi et al. [21] in a retrospective study of 26 cases concluded that on CT, high attenuation areas and calcifications in the solid components of the struma are common findings.

### MRI

The struma has features overlapping with those of malignant ovarian epithelial tumours, as it presents either as a unilateral complex adnexal mass often associated with ascites, or as a multi-cystic mass with solid components and multiple cystic locules. Thick septations, measuring 3–10 mm [13], within the lesion have been described [25] and the peripheral cyst wall measures 7–15 mm in thickness [13].

On MR imaging, the signal intensity of the various solid components varies [7]. The classic MR imaging appearance of struma ovarii includes multiple intra-cystic solid areas, representing thyroid tissue, that are of low signal intensity on T2-weighted images and intermediate signal intensity on T1-weighted images [25]. The cystic spaces, on the other hand, demonstrated both high and low signal intensity on T1- and T2-weighted images [26, 27]. The high-density spaces seen on CT (especially if >90 HU) were of high signal intensity on T1-weighted imaging and low signal intensity on T2-weighted imaging [24]. Some cystic spaces demonstrated low signal intensity on both T1- and T2-weighted images. This pattern of signal intensity on T1- and T2-weighted imaging was found to be due to the thick, highly viscous, gelatinous colloid material in large follicles of the struma [25–28]. Joja et al. [28] stated that the variety of signal intensities seen on MR images in the cystic components depends on the degree of condensation of thyroglobulin and thyroid hormones, and it now recognised that this variable signal intensity is highly characteristic of struma ovarii [25]. In a cohort of 26 cases, Ikeuchi et al. in 2012 [21] concluded that a struma typically presents as a lobulated multicystic lesion with solid components, which frequently includes loculi of low signal intensity on T2-weighted imaging and punctuate foci of high intensity on T1-weighted images.

**Fig. 5:**
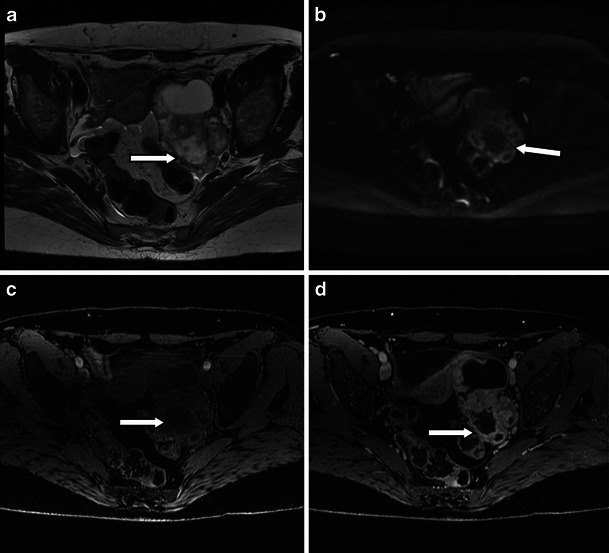
Three-tesla MR images in a 61-year-old patient (same as in Fig. 2) with confirmed benign struma ovarii of the left ovary. An 8-cm left oval-shaped complex multiloculate lesion is seen on the T2-weighted (**a**), diffusion-weighted images with *b* = 1,200 mm/s^2^ (**b**) and T1 fat-saturated images (LAVA) pre-gadolinium (**c**) and post-gadolinium (**d**) within the left hemi-pelvis. The solid portion of the mixed solid and cystic lesion presents with the typical low signal intensity on the T2-weighted image (**a**) and intermediate signal intensity on the T1-weighted image (**c**) (*arrows*). **d** These solid components enhance markedly (*arrow*) which together with the multilobulate surface resemble a “lacy” pattern. **b** This lacy pattern in keeping with solid thyroid tissue is also obvious on the diffusion-weighted images (*arrow*)

**Fig. 6:**
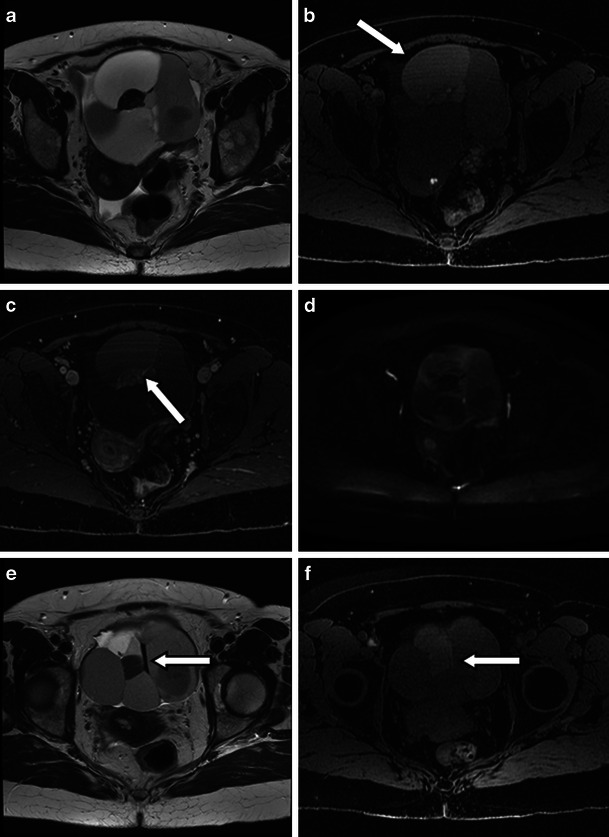
Three-tesla MR images of a predominantly cystic struma ovarii (same patient as Fig. 2a). T2-weighted high-resolution image (**a**), unenhanced (**b**) and enhanced (**c**) T1 fat-saturated LAVA image and diffusion-weighted image *b* = 1,200 mm/s^2^ (**d**) at the same slice level show the multiloculate lesion centrally within the pelvis anterior to the uterus. **a** The cystic components of the struma present with high and low signal intensities on T2-weighted imaging. **b** The cystic areas with high signal intensity on T1 mimic a mucinous epithelial tumour. **c** Contrast-enhanced T1-weighted image illustrates a small solid component presenting with a lacy pattern of enhancement (*arrow*). A linear cystic area in keeping with thick gelatinous fluid demonstrates very low signal on T2-weighted (**e**) and on unenhanced T1-weighted imaging (**f**) (*arrow*)

Imaging features of struma ovarii at 3 T are illustrated in Figs. 5 and 6.

Imaging following an intravenous contrast agent is known to demonstrate marked enhancement of the thick septations and locally thickened wall seen in struma ovarii [25]. The solid components, corresponding microscopically to thyroid tissue, also demonstrate strong enhancement [25] and, together with the multilobulated surface of the struma, gives rise to a “lacy” pattern (Fig. 5).

Intuitively, we assume that the higher spatial resolution achievable at 3 T allows this lacy pattern to be more conspicuous. It goes without saying that the use of a spasmolytic (when no contraindication) and significant patient cooperation are both mandatory in order to achieve the spatial resolution required to enable detection of such textural detail from imaging.

This lacy pattern is also apparent on the diffusion-weighted images: the hyperintense solid components demonstrating restricted diffusion are interspersed with cystic areas (either hyper- or hypointense on T2-weighted imaging) showing increased diffusion (Figs. 5 and 6). From our own experience in a small cohort (unpublished data), apparent diffusion coefficient (ADC) values in the cystic areas of a benign struma are high, while the solid areas present with ADC values within the benign range. The lowest ADC values in our small benign struma cohort of three cases reached up to 1.2 × 10^-3^ mm/s^2^.

Although MR features of struma ovarii overlap with those of other epithelial ovarian lesions, when MR imaging shows a unilateral complex mass with a multilobulate surface and thickened septa, is composed of multiple cysts of variable signal intensity (in keeping with thick viscous colloid fluid) and demonstrates intensely or moderately enhancing solid components with a “lace appearance”, struma ovarii should be included in the differential diagnosis with high probability.

## Unusual imaging findings of struma ovarii

### Cystic struma ovarii

**Fig. 7:**
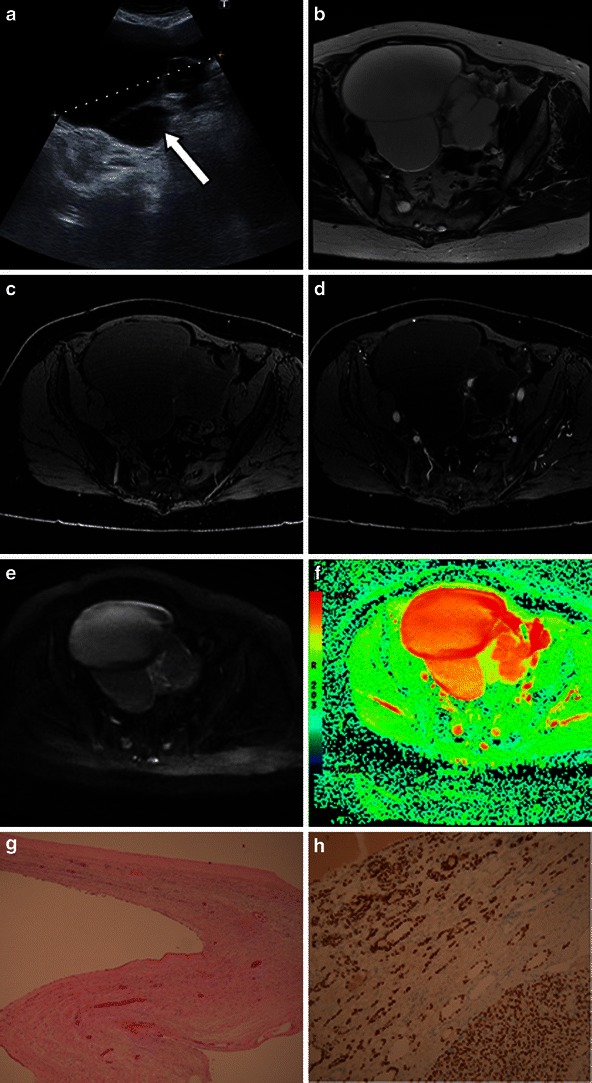
Benign cystic struma ovarii in an 81-year-old woman, presenting with abdominal swelling and discomfort and normal CA125. **a** Ultrasound shows thick septations (*arrow*) within a cystic lesion. Three-tesla T2-weighted high-resolution images (**b**), unenhanced (**c**) and enhanced (**d**) T1 fat-saturated LAVA, diffusion-weighted images *b* = 1,200 mm/s^2^ (**e**) and corresponding ADC map (high ADC values shown in *red*) (**f**) through the same slice are shown. The case macroscopically mimicked a cystadenoma. **g** On histological examination, however, although the cyst loculi were lined by flattened low columnar indifferent epithelium, there were several small areas where micro- and macro-follicles of thyroid tissue were evident. The diagnosis was confirmed with the aid of positive immunostaining for both thyroglobulin (**h**) and TTF1 antibodies. No other teratomatous component was identified despite review of the tissue blocks

A non-cystic struma usually appears as a soft tissue mass, either as a solitary finding or more commonly against a background of a dermoid cyst. Very rarely struma ovarii falls into the category of cystic struma based on its macroscopic appearance. Yen et al. [29] reported one case of a struma ovarii with features on MR imaging that were indistinguishable from a benign multi-cystic ovarian tumour. Only a few cases of cystic struma ovarii have been reported in the literature [29–32]. On MRI, a cystic struma ovarii resembles a cystic epithelial ovarian lesion (Fig. 7). The diagnosis of a cystic struma ovarii is usually made on histopathology. It is recommended that cystic struma ovarii should be considered when evaluating cystic ovarian tumours whose features are not obviously those of another tumour type. If this is the case, a careful search for thyroid follicles should be undertaken. Immunohistochemical staining for thyroglobulin in difficult cases is recommended [32].

### Malignant struma ovarii

Malignant transformation of a mature cystic teratoma is a complication, reported to occur in only 1–2 % of the cases [33], usually in the 6th or 7th decade of life. In total, up to 5–10 % of all cases of struma ovarii are reported to be malignant [34–36]. Such rare cases of carcinoma, usually papillary thyroid cancer, within a struma ovarii have been reported [5].

**Fig. 8:**
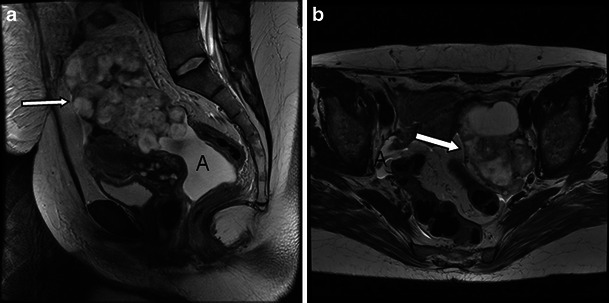
Three-tesla T2 high-resolution images of a benign and malignant struma ovarii for comparison. **a** Sagittal image showing the large mixed solid and cystic malignant struma (*arrow*). **b** Axial plane image through a benign struma shows an equally complex large lesion (*arrow*). **a**, **b** Ascites is present in both the benign and the malignant case (*A*)

**Fig. 9:**
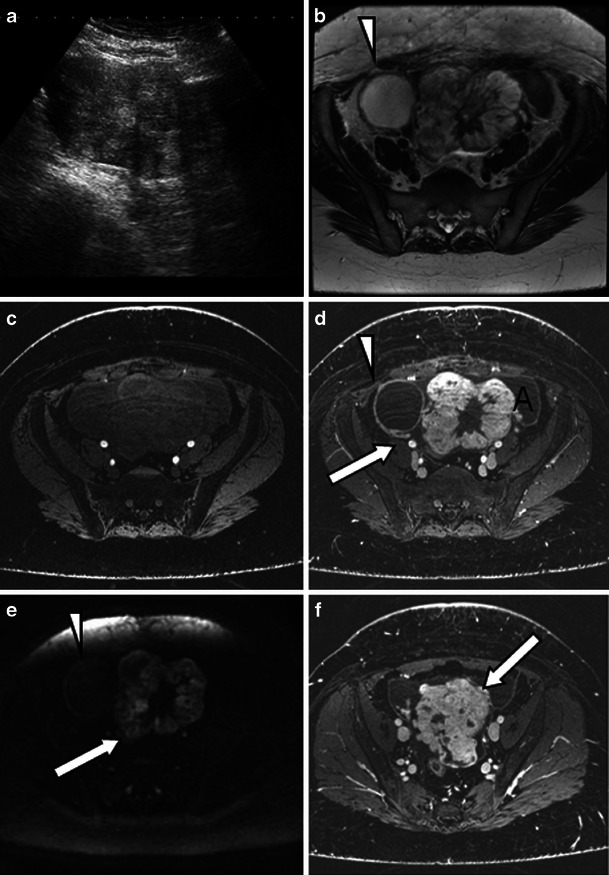
Malignant struma arising from the left ovary in a 40-year-old woman presenting with abdominal distension and lower abdominal pain. CA-125 levels were elevated at 1,075 kU/l (normal value <12 kU/l). **a** Ultrasound shows a mixed solid cystic mass with predominantly solid features. On the 3-T T2-weighted high-resolution (**b**), unenhanced (**c**) and enhanced (**d**, **f**) T1 fat-saturated LAVA and diffusion-weighted images *b* = 1,200 mm/s^2^ (**e**), imaging features resemble a mucinous cystadenocarcinoma. **d**, **e** The lacy pattern caused by the enhancing solid elements (representing thyroid tissue) can be seen on T1 post-contrast imaging (**d**) and diffusion imaging (**e**) might be the only feature to suggest a struma. **d**, **f** Ascites is present (*A*) as well as extracapsular extension which is seen as tumour nodules on the capsular surface (*arrow*). **d**, **e** Some contrast medium uptake is shown in the tissues between the ovary and pelvic sidewall on the right (*arrow*). **b**, **d**, **e** A simple cyst arising from the right ovary does not present with restricted diffusion (*arrowheads*)

The diagnosis of malignant struma ovarii is usually based on histological features of the resected ovary, as no specific imaging features are available to detect malignant struma. However, as malignant struma ovarii are teratomas, the following criteria apply to detect malignancy: CT and MR imaging findings of malignant transformation of ovarian teratoma include invasive growth of large, irregularly marginated soft-tissue components through the tumour wall or irregular soft-tissue components within the tumour [37] (Figs. 8 and 9). In view of the ascites frequently present in benign struma, distinguishing benign from malignant struma is difficult in the absence of extracapsular extension (Fig. 8). The potential future role of diffusion-weighted imaging to distinguish benign from malignant struma remains to be investigated in a large multicentre trial. In our limited experience ADC values in malignant struma overlapped with those of benign struma. However, in this limited cohort of only two cases, the tumour burden in the struma on histopathology was limited.

In many cases of malignant struma, in which the presence of malignancy within the struma was diagnosed on the basis of histological criteria alone, the clinical behaviour was benign [15]. However, distant metastasis has been reported to be a very uncommon feature of struma ovarii. Sites of common metastasis from malignant struma are: the omentum, mesentery, lymph nodes via the peritoneum, liver, brain, bone, lungs and contralateral ovary via a haematogenous route [38, 39]. Metastasis is reported to be possible after removal of the malignant ovarian struma [40]. Such a metastasis to the lumbar spine has been described in a 32-year-old woman [40]. An extreme case was where a distant metastasis was discovered 26 years after initial diagnosis had been reported [12].

In addition, the role of iodine imaging has been emphasised in the detection of recurrent disease after termination of therapy [41].

### Collision tumour

#### Strumal carcinoid

A strumal carcinoid is a unique tumour that is characterised by the presence of both carcinoid and thyroid tissue within a struma ovarii. Clinical manifestations of hyperandrogenism or hyperoestrogenism (8 %) and hyperthyroidism (8 %) have been seen in association with strumal carcinoids. Occurrence of associated carcinoid syndrome is rare [35, 42].

#### Coexistence of Brenner tumour and struma ovarii

Ovarian tumours composed only of Brenner tumour and struma ovarii where both elements are present within the tumour are very rare. Both components of such tumours present with different immunohistochemistry markers [43].

#### Differential diagnosis

Imaging does not allow the differentiation of non-functioning struma ovarii from other cystic masses [5]. When struma ovarii is not associated with hyperthyroidism, the differential diagnosis includes: mature cystic teratoma without fatty tissue, cystadenoma or cystadenocarcinoma, endometriosis, tubo-ovarian abscess and metastatic tumour, because the images of such lesions may resemble those of struma ovarii [5].

Mucinous cyst should be considered when the lesion presents with high signal intensity locules on T1-weighted imaging, typical of mucinous content, and associated septations, but such features are also typical of struma. Struma ovarii is generally a much more complex lesion compared with mucinous cystadenoma. In a cystadenoma, lacy pattern contrast enhancement is not present. Both mucinous cystadenoma as well as struma present with locules containing hyperintense content on T1, representing the mucinous material in the first and thyroglobulin in the latter [28]. The very low signal on T1 and T2 of the gelatinous colloid in the struma may be overlapping with features representing focal haemorrhage within a mucinous tumour, but might be more in favour of struma.

Endometriotic cysts present with very low SI on T2-weighted imaging, while the signal intensity on T1-weighted imaging is usually very high. Hence, discrimination from struma ovarii is generally not difficult.

A tubo-ovarian abscess presents with typical clinical signs of pain, fever and hyperleucocystosis, discrepant to the struma’s often more insidious presentation.

The most challenging differential diagnosis is with ovarian cancer. Malignant epithelial cysts serous as well as mucinous type can present with very complex features, resembling the architecture of the struma’s lacy pattern. Moreover, when haemorrhage is present in ovarian cystadenocarcinoma, this may present with similar features to the colloid in struma. In the absence of omental disease, the differential with malignant epithelial cyst remains challenging.

#### Treatment and impact of preoperative diagnosis in struma ovarii

Treatment for benign struma is surgical resection. Hence, for women wishing to retain fertility, unilateral salpingo-oophorectomy may still be a feasible option in the absence of extracapsular extension and distant metastases [44]. As the few reported imaging features of ovarian struma overlap with those of other lesions, such as ovarian carcinoma, it becomes all the more relevant to be aware of contemporary imaging findings, including state-of-the-art MRI and diffusion-weighted imaging, in order to attempt preoperative diagnosis in these challenging and complex lesions. Failure to characterise this rare and usually benign mass as benign may lead to surgical overtreatment when ovarian cancer type surgery (including bilateral salpingo-oophorectomy, hysterectomy, omentectomy and occasionally appendectomy) is performed.

An adjuvant treatment technique that has been suggested for residual, metastatic or recurrent disease of malignant struma is radioiodine therapy, which has been reported to result in favourable outcomes [4]. In patients presenting with multiple metastatic lesions, or for those who absorb radioiodine poorly, external beam radiation has been proposed [39].

## Summary

At present, the role of imaging in the preoperative characterisation of struma ovarii is limited. The small number of cases of struma ovarii reported in the literature is a significant limitation. Further study in a larger cohort of these rare ovarian tumours through a multicentre collaboration would be useful to see whether there is a novel way of imaging to facilitate preoperative diagnosis and to improve distinction between benign from malignant struma ovarii. Such future research needs to establish the potential role of novel imaging techniques, such as diffusion-weighted imaging, in this rare tumour. Intuitively, high field, high spatial resolution, 3-T imaging allows higher conspicuity of the typical lacy pattern of the struma post-contrast, but this remains to be investigated. Because 95 % of struma ovarii are benign and can occur in premenopausal women, and because the surgery performed is quite different for benign and malignant ovarian tumours, such preoperative radiological diagnosis, however challenging, is very important.
